# Structural and functional insight into the effect of AFF4 dimerization on activation of HIV-1 proviral transcription

**DOI:** 10.1038/s41421-020-0142-6

**Published:** 2020-02-18

**Authors:** Dan Tang, Chunjing Chen, Ga Liao, Jiaming Liu, Banghua Liao, QingQing Huang, Qianqian Chen, Jiahui Zhao, Hui Jiang, Jinsong Duan, Jin Huang, Kunjie Wang, Jiawei Wang, Cuiyan Zhou, Wendan Chu, Wenqi Li, Bo Sun, Zhonghan Li, Lunzhi Dai, Xianghui Fu, Wei Cheng, Yuhua Xue, Shiqian Qi

**Affiliations:** 1 Department of Urology, State Key Laboratory of Biotherapy, West China Hospital, College of Life Sciences, Sichuan University, and Collaborative Innovation Center for Biotherapy, Chengdu, Sichuan 610041 China; 2 School of Pharmaceutical Sciences, Xiamen University, Xiamen, Fujian 361102 China; 3 State Key Laboratory of Oral Diseases, National Clinical Research Center for Oral Diseases, Chinese Academy of Medical Sciences Research Unit of Oral Carcinogenesis and Management, West China Hospital of Stomatology, Sichuan University, Chengdu, Sichuan 610041 China; 4 State Key Laboratory of Membrane Biology, Beijing Advanced Innovation Center for Structural Biology, School of Life Sciences, Tsinghua University, Beijing, 100084 China; 5 School of Biomedicine in Tsinghua University, National Protein Science Facility, Beijing, 100084 China; 6 Shanghai Synchrotron Radiation Facility, Shanghai Advanced Research Institute, Chinese Academy of Sciences, Shanghai, 201204 China

**Keywords:** X-ray crystallography, Transcription

## Abstract

Super elongation complex (SEC) is a positive regulator of RNA polymerase II, which is required for HIV-1 proviral transcription. AFF1/4 is the scaffold protein that recruits other components of SEC and forms dimer depending on its THD domain (TPRL with Handle Region Dimerization Domain). Here we report the crystal structure of the human AFF4-THD at the resolution of 2.4 Å. The α4, α5, and α6 of one AFF4-THD mediate the formation of a dimer and pack tightly against the equivalent part of the second molecule in the dimer of AFF-THD. Mutagenesis analysis revealed that single mutations of either Phe1014 or Tyr1096 of AFF4 to alanine impair the formation of the AFF4 dimer. In addition, transactivation assay also indicated that Phe1014 and Tyr1096 of AFF4 are critical to the transactivation activity of AFF4. Interestingly, the corresponding residues Phe1063 and Tyr1145 in AFF1 have an effect on the transactivation of HIV-1 provirus. However, such mutations of AFF1/4 have no effect on the interaction of AFF1/4 with other subunits of the SEC. Together, our data demonstrated that the dimerization of AFF1/4 is essential to transactivation of HIV-1 provirus.

## Introduction

Acquired immune deficiency syndrome is a global threat caused by the human immunodeficiency virus-1 (HIV-1)^1^. Despite progress and widespread application of antiretroviral therapies (ARTs), HIV-1 remains incurable. The major problem of developing an HIV-1 cure lies in the ability of the virus to persist in patients as latent provirus, which had integrated itself into the genome of CD4+ cells. Latent provirus can then later generate new infectious virus quickly should ARTs fail^2^.

To thoroughly clear HIV-1, a therapeutic approach known as “shock and kill” is studied intensively. This strategy aims to first reactivate the latent provirus, then suppress the transcription of reactivated virus by ARTs while eliminating the exposed virus-producing cells^3^. In addition, an opposite approach called “block-and-lock” provides an alternative for a functional cure of HIV, in which agents are used to establish a state of deep latency that prevents provirus from reactivation^3,4^.

Proviral reactivation is regulated transcriptionally, of which each step, including initiation, elongation, and termination, is monitored by host factors known as checkpoints^5,6^. Once recruited to the HIV-1 proviral DNA promoter, Pol II initiates the transcription. However, a pause occurs after a transcript of 30–50 nucleotides containing the transactivation response (TAR) RNA^6^. To release Pol II from this pause, HIV-1 trans-activator protein (Tat) recruits the super elongation complex (SEC) to the TAR RNA. Subsequently, SEC phosphorylates the C-terminal domain (CTD) of Pol II as well as several negative regulators of Pol II, which clears the pause of transcription elongation of HIV-1 DNA^7,8^.

SEC is composed of the positive elongation factor b (P-TEFb) including cyclin-dependent kinase 9 and Cyclin T (CycT1/2)^9^, the transcription factor ELL1/2^10,11^, the scaffold protein AFF1/4^7,8,10^, and ENL/AF9^7,12^. Tat, the core activator of HIV-1 transcription, is able to simultaneously draw CycT1, AFF1/4, and the TAR RNA together, which facilitates the phosphorylation of the CTD of Pol II by P-TEFb^7,13^. The structure of Tat:P-TEFb:AFF4 reveals that AFF4 (32–67) embeds itself in a hydrophobic groove on the surface of CycT1 and that Tat packs against the two cyclin-fold domains, the Tat-TAR recognition motif of CycT1 and the second helix of AFF4^14–17^.

AFF1/4 is an intrinsic disordered scaffold protein that ties together the other components of the SEC. Intensive studies have uncovered the basis on which AFF1/4 binds to its partners in the SEC. The 60 N-terminal residues of AFF4 interact with P-TEFb by folding into an extended strand followed by two small helices^14–17^. AFF4 (300–350), known as ELLbow, binds to the Occludin domain of ELL2. The binding region forms a cavity inside the complex, which might be a potential target for transactivation^18^. AFF4 (761–774) along with the ANC1 homology domain of AF9 forms an intermolecular three-stranded β-sheet^19^. Although the three binding sites of AFF4 that bind to its partners in SEC have been well defined, the structure of its C-terminal homology domain (hereafter referred to as THD) of AFF4 has not been solved. The THD of AFF4 was reported to form either homodimer or hetero-dimer with AFF1^20–22^. In vivo assays showed that deletion of THD of AFF4 almost completely block its transactivation activity on HIV-1 provirus^18^. However, it remains elusive whether the THD of AFF4 forms a dimer in vitro and whether the dimerization of AFF4 is critical to transactivation of HIV-1 provirus.

Here we determined the crystal structure of THD of human AFF4 at the resolution of 2.4 Å using Se-SAD phasing. As this manuscript was being prepared, Chen et al. reported the structure of AFF4-THD and indicated that AFF4-THD has higher affinity to RNA than to DNA^23^. This work also found that the mutant (H1090A/Y1096A/ V1097A/F1103A/L1104A) of AFF4 cannot form a dimer; however, the effect of each residue was not investigated. Our crystal structure shows that two hydrophobic cores, with Phe1014 and Tyr1096 located in the centers, respectively, mediate the formation of an AFF4 dimer. Single mutations of either residue (Phe1014, Tyr1096) are able to disrupt the formation of the AFF4 dimer as well as cripple the transactivation of HIV-1 provirus, whereas such mutations have no effect on the interaction of AFF4 with other subunits of SEC.

## Results

### Crystal structure of AFF-THD

**Fig. 1:**
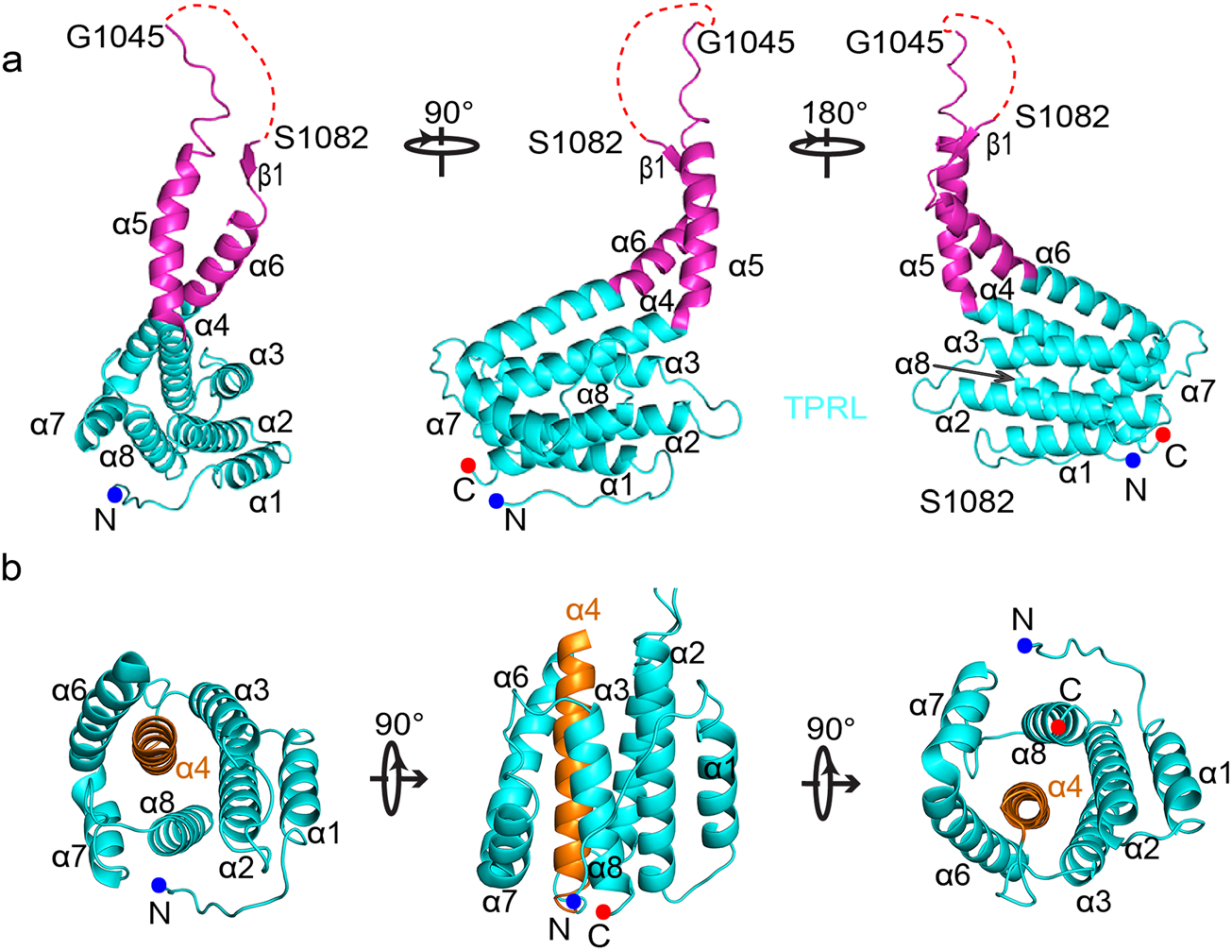
Crystal structure of AFF4-THD. **a** Overview of AFF4-THD. The TPRL domain is colored in cyan, while the Handle Region is colored in magenta. The missing loop (Gly1045-Ser1082) in crystal structure is shown in red dashed line. From left to right, three different views of AFF4-THD domain. The blue and red dots denote the N- and C-terminal of AFF4-THD. **b** Organization of helices in the TPRL domain. From left to right, top view, side view, and bottom view. The α4 is shown in orange.

The crystal structure of human AFF4-THD was determined by Se-SAD, and the structure was refined with native data to 2.4 Å resolution (Supplementary Fig. S1, Table S1). Only one molecule of AFF-THD presents in the asymmetric unit though the AFF-THD was eluted at a volume consistent with dimer from a gel filtration column (Supplementary Table S2). Most residues were localized clearly, except for the highly flexible loop from G1045 to S1082 as well as the very N- and C-terminal residues (Fig. 1a).

The structure of AFF-THD is comprised of eight alpha helices, of which α2, α3, α6 (Ala1116–Ala1120), α7, and α8 constitute a ring with α4 located in the center (Fig. 1b, Supplementary Fig. S2). The α1 helix, with a highly dynamic N-terminal loop standing out of the ring, packs along α2 in the manner of a helix–turn–helix fold (Fig. 1a). Together, those helices mentioned above constitute the tetratrico peptide repeat like domain (TPRL) (Fig. 1a). α5 and the N-terminal portion of α6 (Gln1087 to Asn1102) sticks out of the TPRL domain, which resembles a handle of TPRL domain and is referred to as Handle Region (HR) hereafter (Fig. 1a).

A search of PDB using Dali server with TPRL domain as a query revealed that AFF4-TPRL resembles the tetratrico peptide repeats (TPRs) of Afadin^24^, Apc5^25^, DHR81^26^, and 14–3–3 β protein^27^ with *Z*-scores of 12.2, 11.9, 9.1, and 8.8, respectively. This result is consistent with the published data^23^.

**Fig. 2:**
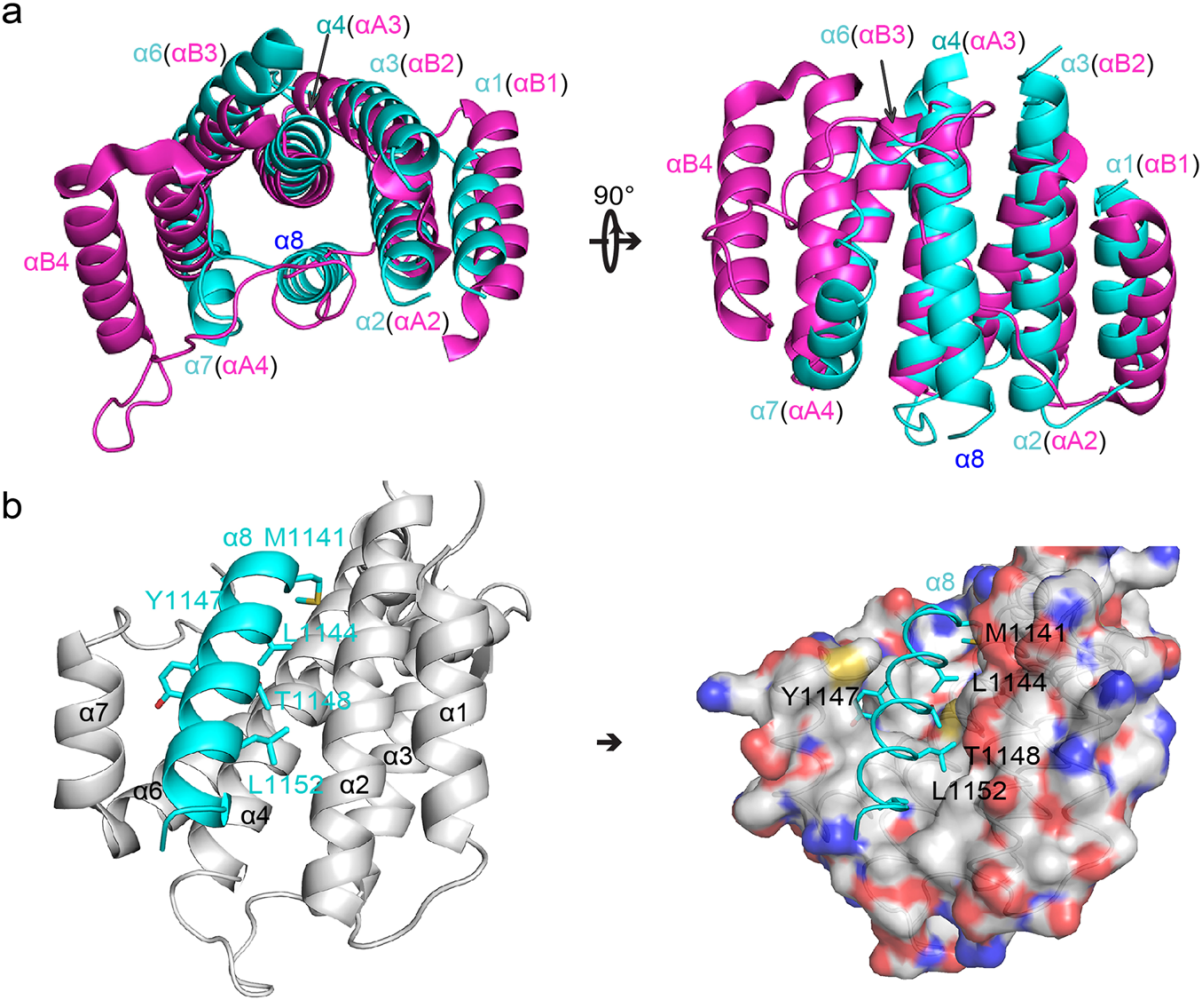
The feature of AFF4-TPRL domain. **a** Comparison of AFF4-TPRL and Afadin-TPR domain shows that the folds are similar but α8 of AFF4 is present in the crescent of AFF4-TPRL. Left: top view. Right: side view. AFF4-TPRL is shown in cyan while Afadin-TPR (5A6C-B) is show in magenta. Helices of AFF4 are labeled as α1–α8, whereas those in Afadin are labeled as αA/αB to show the feature of classic TPR. **b** The hydrophobic groove occupied by α8 is shown as cartoon (left) and surface (right). The helices constituting the groove are shown in gray while α8 is shown in cyan. The key residues of α8 packing into the groove are shown as sticks.

The TPR consists of Helix A and Helix B that arrange in an antiparallel helix–turn–helix^28,29^. In the TPR solenoid, Helix A packs against Helix B as well as Helix A’ of the following TPR, which generates a crescent shape with Helix A located on the concave side (Supplementary Fig. S3). Compared with the TPRs of Afadin, Apc5, DHR81, and 14–3–3 β, we found that α2 and α4 of AFF4-TPRL match well with Helix A, while α1, α3, and α6 are superimposable onto Helix B (Fig. 2a, Supplementary Fig. S4). The α7 and the loop connecting α7 and α8 occupies the position of Helix A (Fig. 2a, b). However, α8, instead presenting on the position of Helix B, takes up the concave groove that is comprised of α2, α4, and α7 (Fig. 2b). Such folding likely facilitates the stabilization of AFF4-TPRL.

Although the sequence of AFF4-TPRL is not predicted as TPRs, AFF4-TPRL arranges in a fold that resembles three TPRs with α8 posing in the concave groove. Hence, we suggest to term this helical repeat as TPRL domain. Intriguingly, both Afadin and Apc5 fold their C-terminal flexible loop into the concave groove, which stabilizes the overall structure. In contrast, AFF4-TPRL contributes its last helix to fill the hydrophobic cleft formed by AFF4-TPRL, a novel protein fold (Fig. 2b).

### Interface of the AFF4-THD dimer

**Fig. 3:**
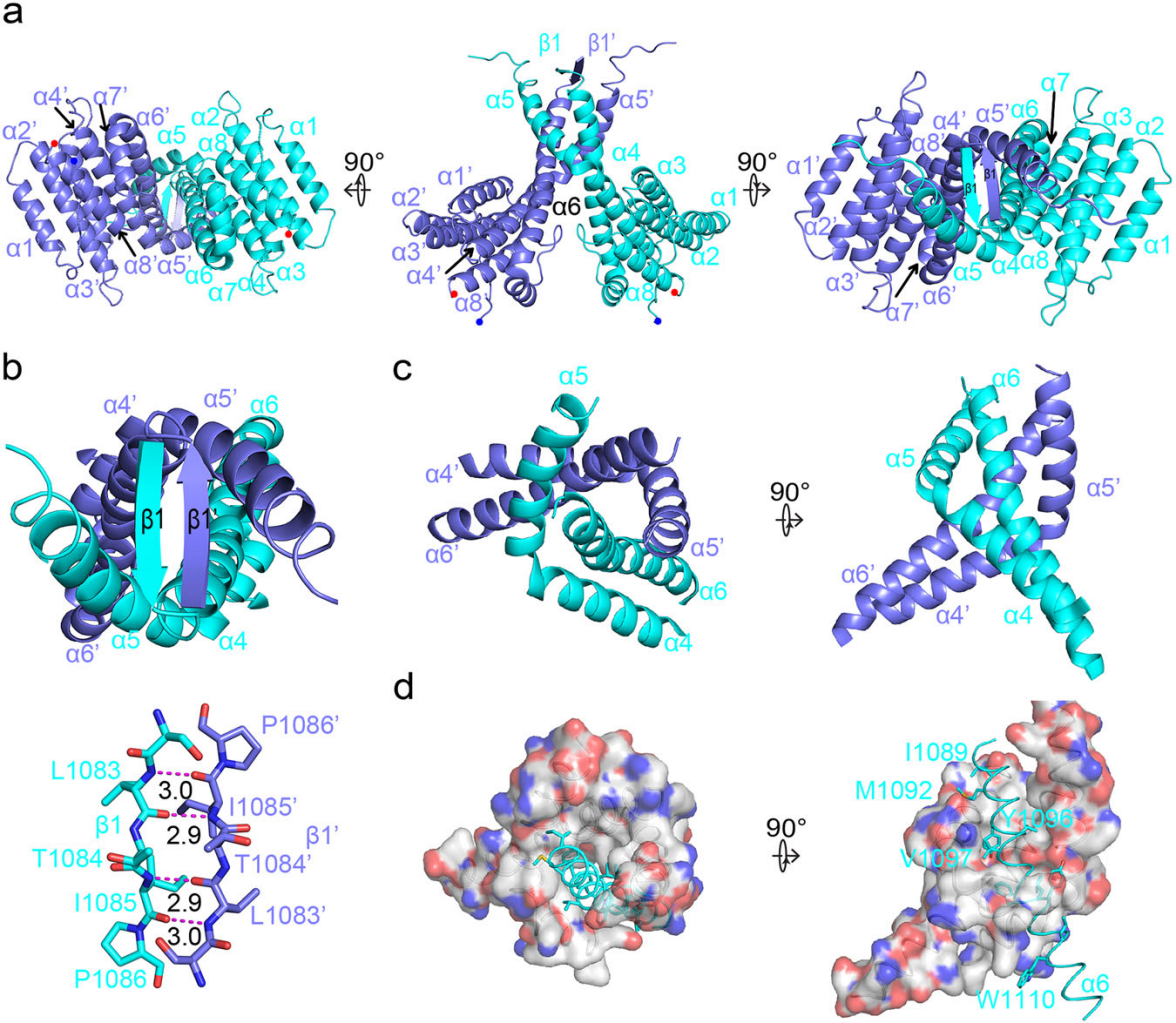
The structure of AFF4-THD dimer. **a** Overview of AFF4-THD dimer. From left to right, bottom view, side view, and top view. One molecule is shown in cyan while the other is shown in slate. The blue and red dots denote the N- and C-terminal of AFF4-THD. **b** The antiparalleled beta-strand is shown. Upper: cartoon. Lower: sticks. The hydrogen bonds are shown in magenta dash line and the length of hydrogen bond is labeled. **c** The helix bundle formed by α5, α6, α5’, and α6’ is shown as cartoon. Left: top view. Right: side view. **d** The hydrophobic groove formed by α5, α5’, and α6’ is shown as surface, while α6 is shown as cartoon with key residues shown as sticks. The orientation of view is same to that in **c**. Left: top view. Right: side view.

Though only one AFF-THD was visualized in the asymmetric unit, analytical gel filtration indicated that AFF-THD is a dimer in solution (Supplementary Table S2). Therefore, we generated a dimer model in Pymol according to I2_1_2_1_2_1_ symmetry (Fig. 3a). As shown in the model, the dimerization of AFF-THD is mediated mainly by α5, and α6 (Ser1082 to T1107), as well as part of α4 (Fig. 3a). The interface has two-fold symmetry that buries a surface area of 3219 Å^2^ in total. To present the structure clearly, we denoted the residues, secondary structures of the second molecule of AFF4-THD, with prime (’). The two HR domains form a four-helix bundle, of which the top forms an intermolecular antiparallel β strand (Fig. 3b). α5, α6, and α5’ constitute a hydrophobic groove in which α6’ tightly packs and is almost buried (Fig. 3c), and vice versa.

**Fig. 4:**
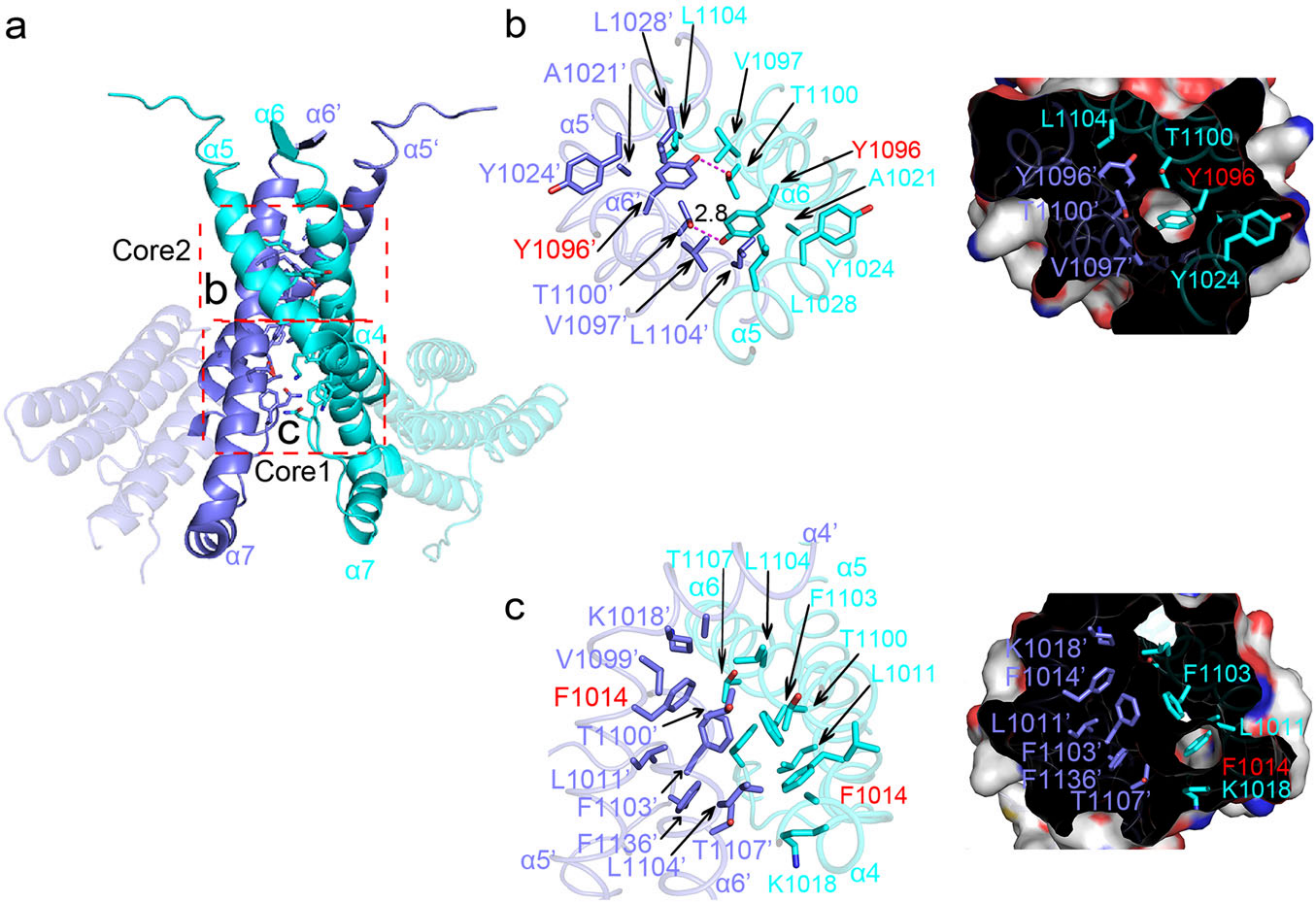
The interface of AFF4-THD dimer. **a** Overview of interface of AFF4-THD dimer. Two molecules of the dimer are colored in cyan and slate, respectively. **b**, **c** Details of Core2 and Core1. Cartoon and surface representation are shown while the key residues are shown in stick model. The residues labeled with “’” represent that these residues are from the second molecule. The labels of F1014 and Y1096 are colored in red. In the surface model, F1014 and Y1096 are shown in hydrophobic pockets.

Residues involved in the dimerization of AFF-THD compose two hydrophobic cores, of which the centers are Phe1014 (Core1) and Y1096 (Core2), respectively (Fig. 4a, Supplementary Fig. S2). Surrounding Phe1014 is a hydrophobic pocket that comprises residues from α4, α5, α6, and α6’, including Leu1011, Val1099, Phe1103, Leu1104’, and the aliphatic part of Lys1018 and Thr1107 (Fig. 4c, Supplementary Fig. S2).

Tyr1096 of α6 forms a 2.8 Å hydrogen bond with Thr1100’ from α6’; however, this polar interaction is buried deeply by the aliphatic or hydrophobic part of Ala1021, Tyr1024, Leu1028, and Thr1100, as well as Tyr1024’, Val1097’, Leu1104’ of α6’ (Fig. 4b, Supplementary Fig. S2).

Thr1100 plays dual roles in the dimerization of AFF-THD: forming a hydrogen bond with Tyr1096’ and donating its γ methyl group to the hydrophobic environment surrounding Tyr1096 (Fig. 4b).

### The hydrophobic cores are required for the dimerization of AFF-THD

**Fig. 5:**
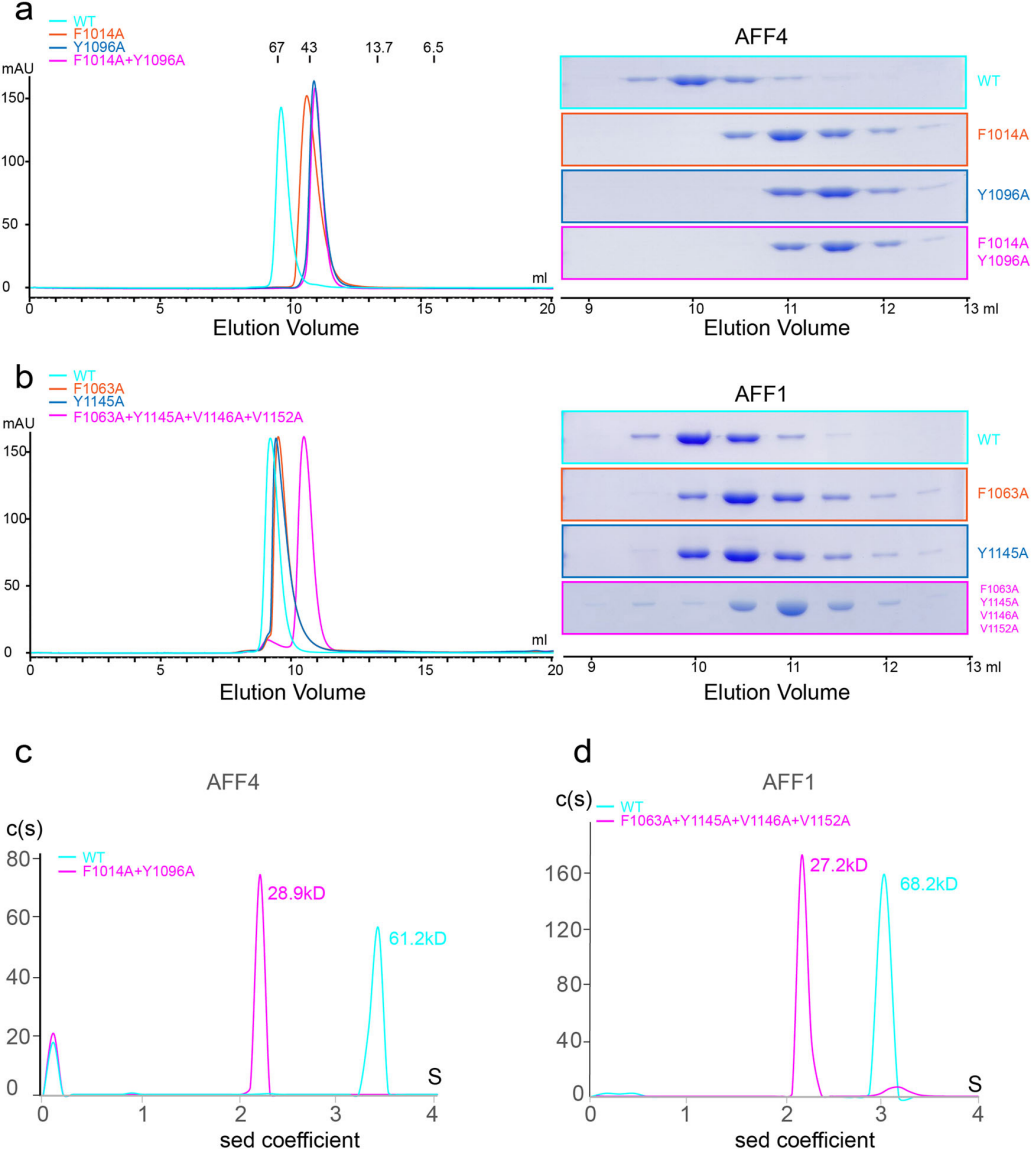
Contribution of hydrophobic cores of AFF4-THD to dimerization. **a**, **b** Analytical gel filtration revealed that the hydrophobic cores are critical to the dimerization of AFF1/4. Left: gel filtration chromatography, the horizontal axis is elution volume, and the vertical axis is UV absorption. The molecular weight standards (kD) are denoted on top of the curves. Right: SDS-PAGE results of peak fractions of corresponding gel filtration chromatography, and the elution volume in gel filtration is denoted below. **a** The curve of wild-type AFF4-THD, AFF4^F1014A^, AFF4^Y1096A^, and AFF4^F1014A+Y1096A^ are colored in cyan, orange, blue, and magenta, respectively. **b** Wild-type AFF1-THD, AFF1^F1063A^, AFF1^Y1145A^, and AFF1^F1063A+Y1145A+V1146A+V1152A^ are colored in cyan, orange, blue, and magenta, respectively. **c**, **d** Sedimentation velocity analytical ultracentrifugation results. Curve of wild type of AFF1/4 are shown in cyan, while the mutations of AFF1/4 are shown in magenta. The calculated molecular weight is denoted on top of these peaks. Horizontal axis: sedimentation coefficient. Vertical axis: continuous sedimentation coefficient distribution.

We applied mutational analysis to explore the role of the two hydrophobic cores. As the interface area is so large, we predicted that a single mutation would not prevent dimerization. Hence, multiple mutants were designed for the purpose of generating monomers of AFF-THD (Supplementary Table S2). The critical residues Phe1014 and Tyr1096 were mutated to Ala and termed as AFF4-THD^F1014A+Y1096A^. The effect of this mutation was assessed by analytical gel filtration, and the elution volume of AFF4-THD^F1014A+Y1096A^ corresponded to the position of monomer (Fig. 5a, Supplementary Table S2). To corroborate this observation, the analytical ultracentrifugation (AUC) experiment was carried out. The sedimentation coefficients of wild-type AFF4-THD and AFF4-THD^F1014A+Y1096A^ were 3.43 and 2.22 S, which correspond to molecular masses of ~61.2 and ~28.9 kD, respectively (Fig. 5c). Together with the results of analytical gel filtration and the theoretical molecular mass of AFF4-THD, AFF4-THD^F1014A+Y1096A^ was demonstrated to be monomer in solution.

We also tested the effect of single mutations of those key residues in AFF4-THD^F1014A+Y1096A^ on the dimerization of AFF4-THD. Surprisingly, and against our prediction, the elution volume of AFF4-THD^F1014A^ from gel filtration corresponded to the molecular mass of ~40 kD, which indicated that AFF4-THD^F1014A^ disrupted the dimer dramatically (Fig. 5a, Supplementary Table S2). Since the effect of single mutation of Phe1014 is strong, we probed the effect of Tyr1096 at the center of Core2. Analytical gel filtration showed that AFF4-THD^Y1096A^ was in monomeric state (Fig. 5a, Supplementary Table S2).

Single mutations of other critical residues in Core2, including Y1024A and S1025A, has little impact on the dimerization of AFF4-THD (Supplementary Table S2). The elution position of AFF4-THD^Val1097A^ from gel filtration shifted to the position between dimer and monomer, which indicated that Val1097A of AFF4-THD weakens the dimer formation instead of destroying the dimer. Mutant of AFF4-THD^T1100A+Y1096F^ did not impact on the formation of the dimer, which indicated that the polar interaction is not essential to dimerization (Supplementary Table S2). Interestingly, the calculated molecular mass of AFF4-THD^Y1096H^ based on analytical gel filtration was 43.5 kD, indicating that the mutant interrupts the formation of AFF4-THD dimer (Supplementary Table S2). The effect of antiparallel β strands was assessed by replacement of this region with a linker composed of 5-glycine. Analytical gel filtration results showed this mutant had little effect on dimerization (Supplementary Table S2).

AFF1-THD shares 44.3% identity with AFF4-THD (Supplementary Fig. S2). Moreover, the key residues that mediate dimerization of THD are conserved between AFF1 and AFF4. AFF1-THD existed as dimer in solution (Fig. 5b, d), which is consistent with its status in vivo^21^. Hence, we investigated the effect of the hydrophobic cores on the dimerization of AFF1-THD. However, the results of analytical gel filtration showed that neither of AFF1-THD^F1063A^, AFF1-THD^Y1145A^, and AFF1-THD^F1063A+Y1145A^ was able to disrupt the formation of AFF1-THD dimer completely, which indicated that these mutants have no obvious effect on the dimerization of AFF1-THD in vitro (Fig. 5b, Supplementary Table S1). To destroy the hydrophobic cores completely, V1146 and V1152, close to Y1145 and F1063, respectively, were mutated into alanine on the basis of AFF1-THD^F1063A+Y1145A^. The elution position of AFF1-THD^F1063A+Y1145A+V1146A+V1152A^ from gel filtration delayed and corresponded to monomer that indicated that this mutant prevents the formation of AFF1-THD dimer. The sedimentation coefficient of AFF1-THD^F1063A+Y1145A+V1146A+V1152A^ based on the result of AUC was 2.12 S, corresponding to a molecular mass of 27.2 kD and indicating that this mutant is monomeric in solution (Fig. 5d). Together, the hydrophobic cores have appreciable effects on dimerization of AFF-1-THD in vitro.

### Dimerization of AFF4 is essential to HIV-1 proviral transactivation

**Fig. 6:**
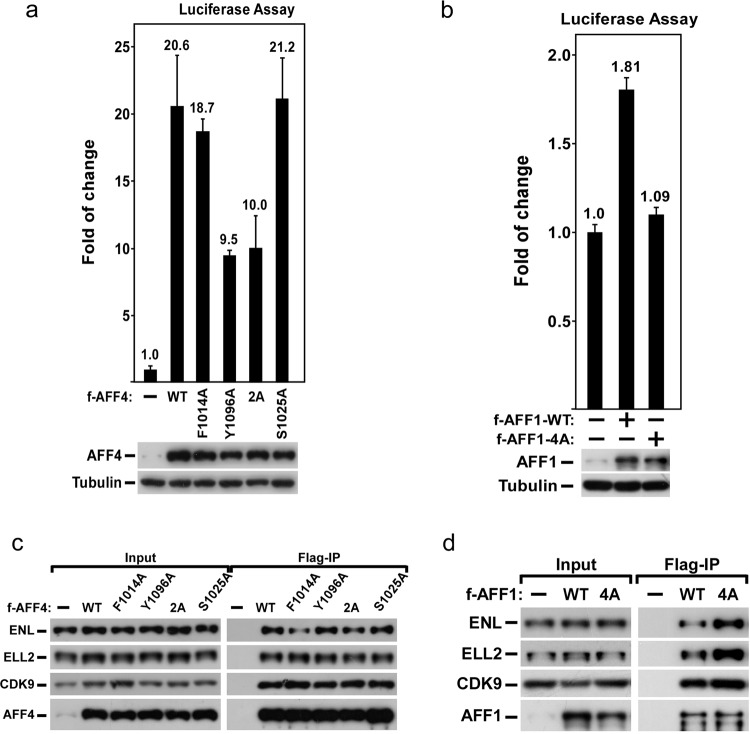
Dimerization of AFF1/4 is essential to Tat-dependent HIV-1 proviral transactivation. **a**, **b** Top boxes: luciferase activities were measured in NH1 cell extracts, in which wild-type or mutated Flag-AFF4/1 was transfected as indicated. The activity in the control groups were artificially set to “1.0.” The error bars represent mean +/– SD. Bottom boxes: western blot analysis of the levels of tested proteins in the transfected NH1 cells as indicated. WT, F1014A, Y1096A, 2A, and S1025A in **a** represent wild-type AFF4, AFF4 ^F1014A^, AFF4 ^Y1096A^, AFF4^F1014+Y1096A^, and AFF4^S1025A^, respectively. AFF1–4A in **b** represents AFF1^F1063A+Y1145A+V1146A+V1152A^. **c**, **d** Nuclear extracts (NE) were prepared from NH1 cells transfected with the indicated plasmids and subjected to immunoprecipitation (IP) with anti-Flag AFF4 (**c**) or anti-Flag AFF1 (**d**). The NE inputs and IP eluates were examined by immunoblotting for the presence of the tested proteins indicated on the left. The labels in **c**, **d** are same to that in **a**, **b**.

To test the role of dimerization of AFF1/4 in HIV-1 proviral transactivation, a luciferase reporter assay was performed in HeLa-based NH1 cells, in which the HIV-LTR-luciferase is integrated into the genome. Overexpression of AFF4 transactivates HIV-1 provirus by 20-fold in the NH1 cell, consistent with previous work^18^. However, overexpression of AFF4^F1014A^ had ~10% less proviral transactivation activity than wild type, and AFF4^Y1096A^ had onefold less proviral transactivation activity than wild type (Fig. 6a). Moreover, the mutant combining F1014 and Y1096 had similar activity in HIV-1 provirus transactivation as AFF4^Y1096A^ (Fig. 6a). As a control, AFF4^S1025A^, having little effect on the dimerization of AFF4, showed similar activity in HIV-1 proviral transactivation as that of wild-type AFF4. These observations are consistent with the fact that both AFF4^Y1096A^ and AFF4^F1014A+Y1096A^ disrupted dimerization of AFF4-THD dramatically (Fig. 5a, c). Together, the dimerization of AFF4 is critical to HIV-1 proviral transactivation.

To determine whether the dimerization of AFF1 is required for HIV-1 provirus transactivation, the most effective mutant, AFF1^F1063A+Y1145A+V1146A+V1152A^, was overexpressed in NH1 cells in the luciferase reporter assay. As shown in Fig. 6b, AFF1^F1063A+Y1145A+V1146A+V1152A^ had onefold less proviral transactivation activity than wild-type AFF1, indicating that AFF1^F1063A+Y1145A+V1146A+V1152A^ lost the activity of HIV-1 provirus transactivation, compared to wild-type AFF1 (Fig. 6b).

To rule out the possibility that mutants of AFF1/4 disrupt their binding to the components of SEC, an in vivo pull-down was performed. AFF4^F1014A^, AFF4^Y1096A^ and AFF4^F1014+Y1096^ bind to other subunits of SEC as strong as the wild type (Fig. 6c). A similar result was seen with AFF1^F1063A+Y1145A+V1146A+V1152A^ (Fig. 6d).

Together, these findings support that dimerization of AFF4 is essential to HIV-1 proviral transactivation and is not involved in the binding to other subunits of SEC.

## Discussion

AFF1/4-THD is indispensable for HIV-1 provirus transactivation. Previous structural studies have shown how AFF4 recruits other subunits of SEC, including P-TEFb, ENL/AF9, and ELL2. A less defined function of AFF is how the THD mediates either homodimerization or heterodimerization of AFF1/4. In this work, extensive structural analysis, biochemical assays, proviral transactivation assays, and in vivo pull-downs clarify the structural basis of AFF1/4 dimerization as well as the functional importance of AFF1/4 dimerization for transactivation of HIV-1 provirus.

Although the base region of AFF4-THD resembles a TPR domain, the overall structure of AFF4-THD represents a novel protein fold (Fig. 2a, b). The α5 and N-terminal part of α6, sticking out of the base region, form the handle region of THD that mediates the dimerization of THD. Thus we suggest that the C-terminal homology domains of AFF family be referred to as THD (TPRL with Handle motif Dimerization Domain), which represents its structural feature as well as functional characterization. Such a novel protein fold that resembles TPR domain provides a new paradigm for protein design de novo^26,30^. TPR domains mediate protein–protein interactions with their concave face in a wide range of signal pathways^29^. The face presented in the AFF4-THD is occupied by α8 (Fig. 2a), which is too critical for folding to be deleted (data not shown). However, it is tempting to speculate that AFF4-THD can release the α8 and bind to other partners in some special conditions. AFF4-THD had also been indicated to bind to G-quadruplex in vitro; however, functional assays are required to support this conclusion^23^.

Dimerization of AFF4-THD is mainly mediated by two hydrophobic cores located on α4, α5, and α6. Taking into account the molecular weight of AFF-THD, the area of interface, summing up to 3219 Å^2^, is pretty large. However, two central residues of the two hydrophobic cores, Phe1014 and Tyr1096, account for dimer formation. Alanine mutation of either the two residues is able to disrupt the dimer of AFF4. Likewise, Phe1063 and Tyr1145 in AFF1, the corresponding residues of Phe1014 and Tyr1096 of AFF4, have appreciable effect on the dimerization of AFF1-THD.

AFF1/4 is required for HIV-1 proviral transactivation. A single mutation of either Phe1014 or Tyr1096 is able to cripple its activity of HIV-1 proviral transactivation. These observations illustrate the functional importance of dimerization of AFF1/4 in HIV-1 transactivation. However, deletion of THD of AFF4 abrogates its activity of HIV-1 proviral transactivation^18^. Hence, it is possible that AFF4-THD might have other function in HIV-1 proviral transactivation besides dimerization, for example, binding to other factors (nucleic acids or protein) that are required for transactivation^23^.

Furthermore, Phe1014 of AFF4 has been reported to be mutated into leucine in lung cancer and bladder urothelial carcinoma, which indicates that dimerization of AFF4 also plays an important role in the progress of carcinoma^31^. Thus the mutation of key residues on AFF4 dimer interface might be used as indicators of carcinoma development.

Many strategies have been developed to cure HIV-1 infection, including “lock and kill” and “block-and-lock”^3,4^. Both approaches call for thorough understanding of the mechanism of HIV-1 proviral transactivation. Didehydro-cortistatin A is able to prohibit ongoing viremia during ART so that the HIV-1 promoter is locked in deep latency^4^. Here, we found dimerization of AFF1/4 is critical to transcriptional reactivation of HIV-1 provirus, which might provide a potential therapeutic target for “block-and-lock” strategy. Taken the conservation of AFF1/4-THD into consideration, it would be of interest to develop an antibody that is able to block dimerization of both AFF1 and AFF4 to suppress HIV-1 proviral transactivation in the short term.

## Materials and methods

### Antibodies and cell lines

The anti-ENL (A302-267A), anti-AFF1(AF4) (A302-345A), and anti-ELL2 (A302-505A) antibodies were purchased from Bethyl Laboratories. The antibodies against α-tubulin (T9026) and Flag (F3165) were obtained from Sigma. The anti-AFF4 (14662-1-AP) was purchased from Proteintech. Rabbit polyclonal antibodies against AF9 have been described previously^32^.

All the cell lines used in this study were either purchased from American Type Culture Collection (ATCC, Manassas, VA) or described before^33^ or generated in our laboratory.

### Cloning and protein purification

cDNA of human AFF4-THD (902–1163) was subcloned into pHis-parallel2 with a C-terminal His_6_-tag, and DNAs for AFF1-THD (953–1210) were subcloned into pHis-parallel2 with an N-terminal His_6_-tag that can be removed by tobacco etch virus (TEV) protease. Full-length wild-type AFF1/4 were subcloned into pRK5. The plasmids expressing mutant versions of AFF1/4 were generated by PCR mutagenesis. The mutant constructs were verified by DNA sequencing. All proteins used here were expressed in *Escherichia coli* BL21 (DE3) cells. After induction with 0.2 mM IPTG overnight at 16 °C, the bacteria were pelleted by centrifugation at 4000 × *g* for 10 min. The pellets were lysed in 25 mM Tris-HCl pH 8.0, 150 mM NaCl, 0.5 mM TCEP-HCl, and 1 mM phenylmethanesulfonylfluoride (PMSF) by French Press. The lysate was then centrifuged at 25,000 × *g* for 30 min at 4 °C. The supernatants of AFF1/4 were loaded onto Ni-NTA resin at 4 °C; target proteins were eluted with 25 mM Tris-HCl pH 8.0, 150 mM NaCl, 0.5 mM TCEP-HCl, and 250 mM Immidazole pH 8.0; and the eluates were diluted 5-fold with 25 mM Tris-HCl pH 7.0 and applied to a Hi-Trap SP HP column. Peak fractions were pooled and digested with TEV protease at 4 °C overnight. TEV and His_6_-tag were removed by loading the solution onto Ni-NTA. Target proteins were further purified on a Superdex 200 10/300 column equilibrated with 25 mM Tris-HCl pH 8.0, 150 mM NaCl, and 2 mM dithiothreitol (DTT). The peak fractions were pooled and flash-frozen in liquid N_2_ for storage. Selenomethionyl (Se-Met) protein was expressed in *E. coli* BL21(DE3). The cells grew in M9 minimal medium supplemented with 5% LB medium. In all, 0.2 mM IPTG and 50 mg selenomethionine were added into 1 liter culture when the OD_600_ reached 1.0. Cells were pelleted by centrifugation at 4000 × *g* for 10 min after overnight induction at 16 °C. Se-Met AFF4-THD was prepared as above.

### Crystallization of the AFF4-THD

The purified AFF4-THD was concentrated to 8 mg/ml with a 10-kD cut-off centrifugal filter (Millipore). Crystals were grown by hanging-drop vapor diffusion at 18 °C. The protein solution was mixed with well buffer containing 15% PEG3350, 0.3 M sodium citrate tri-basic, and 0.1 M sodium citrate–citric acid pH 4.5. Crystals appeared in 24 h and grew to full size in 2–3 days. Crystals were flash-frozen with liquid N_2_ in well buffer. Se-Met crystals were grown in the same condition as native crystals. Crystals were screened on BL17U, BL18U, and BL19U at Shanghai Synchrotron Radiation Facility (SSRF)^34^. Native data and Se-Met data were collected on BL18U and BL19U at SSRF, respectively. Native crystals diffracted to 2.4 Å, and data were collected at a wavelength of 1.0000 Å. All data sets were processed with HKL2000 (HKL Research). The statistics are shown in Supplementary Table S1. The phase is determined by Se-SAD using SHELX^35^. Model building and refinement were completed with Phenix and Coot^36,37^.

### Analytical gel filtration assay

Wild type and mutants of AFF1/4-THD were purified according to the protocol aforementioned. Target proteins were applied on Superdex 75 10/300 increase column (GE) in 25 mM Tris pH 8.0, 150 mM NaCl, and 2 mM DTT. The concentration and injection volume were 6 mg/ml and 100 μl, respectively. The weight average molecular weight of target protein was calculated by *Y* = − 0.1725*X* + 5.2599 (R^2^ = 0.99669, *Y* = lgMw, *X* = elution volume).

### Analytical ultracentrifugation

Analytical ultracentrifugation sedimentation experiments were carried out at 20 °C in an XL-I analytical ultracentrifuge (Beckman–Coulter) equipped with Rayleigh interference detection (655 nm). BTN3A1 B30.2 (400 ml, 27 mM, 54 mM, 160 mM, 240 mM, respectively) were centrifuged at 50,000 rpm for 8 h in an An50Ti rotorusing 12 mm double-sector aluminum center pieces. All samples were prepared in the interaction buffer (25 mM Tris pH 8.0, 150 mM NaCl, 2 mM DTT). Interference profiles were recorded every 6 min. Data analysis was conducted with the software Sedfit 11.

### Luciferase reporter assay

For luciferase reporter assay, HeLa-based NH1 cells was used, in which the HIV-LTR-luciferase is integrated into the genome. In all, 2 × 10^5^ HeLa-based NH1 cells^38^ in the 6-well plates were transfected in triplicate with the transfection agent PEI at a ratio of 1:3 (plasmid mass: PEI volume) by plasmids expressing wild-type or mutant FLAG-AFF4 or FLAG-AFF1 (2 μg). Forty-eight hours after transfection, the cells were harvested and lysed in 1× Reporter Lysis Buffer (E1501 Promega), followed by centrifugation at 20,800 × *g* for 1 min. Lysate were normalized based on the quantity of α-tubulin that they contained. Luciferase activities in the supernatant were measured using the Luciferase Assay System (E1501 Promega) on a Glomax Discover System (GM3000, Promega).

### Immunoprecipitation assay

The immunoprecipitation assay was performed essentially as described^7^ with minor modifications. Briefly, for anti-Flag immunoprecipitation, nuclear extracts prepared from NH1 cells transfected with the indicated expressing constructs were incubated with anti-Flag agarose beads (Sigma) for 2 h before washing and elution. After incubation, the beads were washed extensively with buffer D0.3 (20 mM HEPES-KOH pH 7.9, 15% glycerol, 0.2 mM EDTA, 0.2% NP-40, 1 mM DTT, 1 mM PMSF, and 0.3 M KCl), eluted with 0.1 M glycine pH 2.5, and analyzed by western blotting with the indicated antibodies.

### Accession number

Coordinates and structure factor of the structure reported here have been deposited into the Protein Data Bank with PDB Code: 6K7P.

## Supplementary information

supplemental material

## Supplementary information

**Supplementary Information** accompanies the paper at (10.1038/s41421-020-0142-6).
