# Supplementary material for: Structural and functional insight into the effect of AFF4 dimerization on activation of HIV-1 proviral transcription
Source: Cell Discov. 2020 Feb 18;6:7. doi: 10.1038/s41421-020-0142-6 (PMC7026398; doi:10.1038/s41421-020-0142-6)
Supplement: Supplementary file 1 — supplemental material [file 41421_2020_142_MOESM1_ESM.pdf]

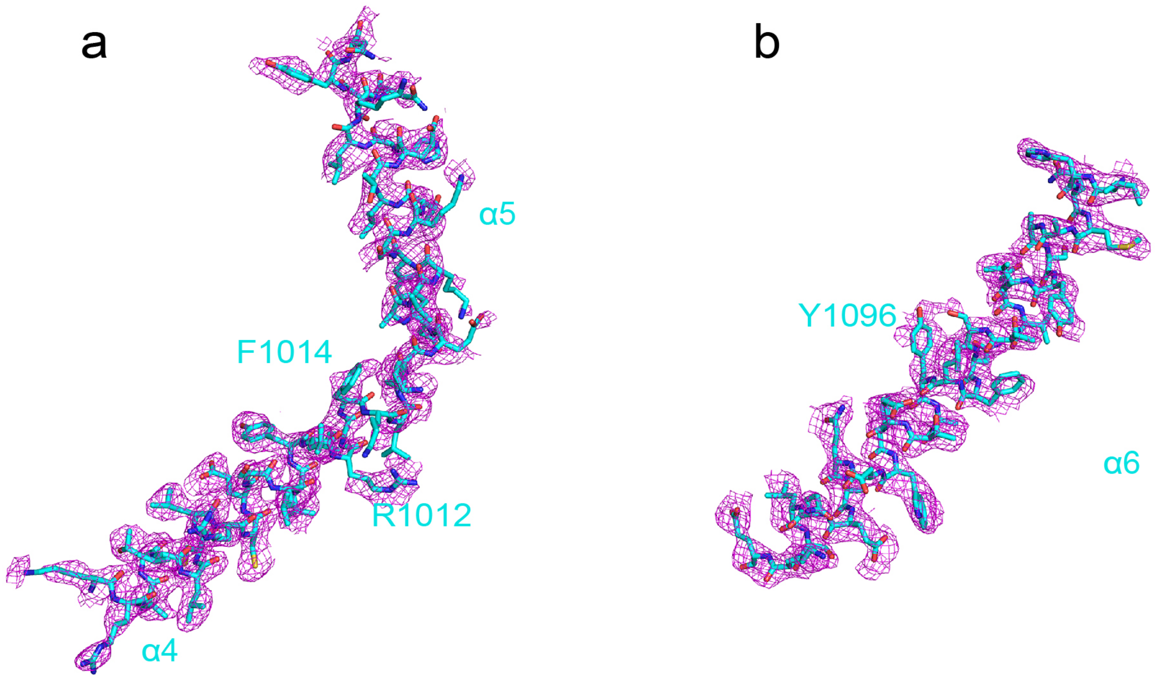

Figure S1

```
                                                                       α1
AFF1_HUMAN      ----------SSGD--TANPFPVPSLPNGNSKPGKPQVKFD--KQQADLHMREAKKMKQK      977
AFF4_Danio      ----------KSS--KSENPVSVPPLSE--GSKSRSKLLFEDRVHSADHYLQEAKKLKHN      849
AFF4_Xenopus    ----------KSANSTAAVAFI--PPID--NKSQRAKLVFEDRIHSADHYLQEAKKLKHN      927
AFF4_MOUSE      ----------KAPNSSSNCPPSTPTSES--SKPRRTKLAFDDRNYSADHYLQEAKKLKHN      925
AFF4_RAT        ----------KAPNSSSNCPPSTPTPDS--SKPRRTKLAFDDRNYSADHYLQEAKKLKHN      915
AFF4_HUMAN      ----------KAPSSSSNCPPSAPTLDS--SKPRRTKLVFDDRNYSADHYLQEAKKLKHN      928
AFF4_BOVIN      ----------KAPNSSSNCPPSAPTPDA--SKPRRTKLAFDDRNYSADHYLQEAKKLKHN      929
AFF2_HUMAN      ITSTITTGLMDSSHLEMTSWAALPLLSSSSTNVRRPKLTFDDSVHNADYYMQEAKKLKHK     1085
AFF3_HUMAN      ------NIPLHKSRPQTKPWSP---GSNGHRDCKRQKLVFDDMPRSADYFMQEAKRMKHK      990

                α1       α2                         α3
AFF1_HUMAN      AELMTDRVGKAFKYLEAVLSFIECGIATESESQSSKSAYSVYSETVDLIKFIMSLKSFSD     1037
AFF4_Danio      ADALMDRFEKAVYYLDAVVSFIECGNALEKNAQEAKSPFPMYAETVELIKYTMKLKSYMA      909
AFF4_Xenopus    ADALSDRFEKAVYYLDAVVSFIECGNALEKSVQESKSPFPMYSETVELIKYTMKLKSSAA      987
AFF4_MOUSE      ADALSDRFEKAVYYLDAVVSFIECGNALEKNAQESKSPFPMYSDTVELIKYTMKLKNYLA      985
AFF4_RAT        ADALSDRFEKAVYYLDAVVSFIECGNALEKNAQESKSPFPMYSDTVELIKYTMKLKNYLA      975
AFF4_HUMAN      ADALSDRFEKAVYYLDAVVSFIECGNALEKNAQESKSPFPMYSETVDLIKYTMKLKNYLA      988
AFF4_BOVIN      ADALSDRFEKAVYYLDAVVSFIECGNALEKNAQESKSPFLMYSETVELIKYTMKLKNYLA      989
AFF2_HUMAN      ADALFEKFGKAVNYADAALSFTECGNAMERDPLEAKSPYTMYSETVELLRYAMRLKNFAS     1145
AFF3_HUMAN      ADAMVEKFGKALNYAEAALSFIECGNAMEQGPMESKSPYTMYSETVELIRYAMRLKTHSG     1050

                         α4                        α5
AFF1_HUMAN      ATAPTQEKIFAVLCMRCQSILNMAMFRCKKDIAIKYSRTLNKHFES-SSKVAQAPSPCIA     1096
AFF4_Danio      PDATSQDKRLAVLCLRCQALLYLRLFKLRKESALKYSKTLTEHLKN-SLSNTQAPSPGVA      968
AFF4_Xenopus    PDATAADKRLGVLCLRCQSLLYLRLFKLKKESALKYSKTLTEHEEQ-CSCNSQAPSPGMG     1046
AFF4_MOUSE      PDATAADKRLTVLCLRCQSLLYLRLFKLKKENALKYSKTLTEHLKN-SYSNSQAPSPGLG     1044
AFF4_RAT        PDATAADKRLTVLCLRCQSLLYLRLFKLKKENALKYSKTLTEHLKN-SYSNSQAPSPGLG     1034
AFF4_HUMAN      PDATAADKRLTVLCLRCESLLYLRLFKLKKENALKYSKTLTEHLKN-SYNNSQAPSPGLG     1047
AFF4_BOVIN      PDATAADKRLTVLCLRCQSLLYLRLFKLKKETALKYSKTLTEHLKN-SYNNSQAPSPGLG     1048
AFF2_HUMAN      PLASDGDKKLAVLCYRCLSLLYLRMFKLKKDHAMKYSRSLMEYFKQNASKVAQIPSPWVS     1205
AFF3_HUMAN      PNATPEDKQLAALCYRCLALLYWRMFRLKRDHAVKYSKALIDYFKN-SSKAAQAPSPWGA     1109
                                          *

                                                        β1        α6
AFF1_HUMAN      S---TGTPSPLSPMPSPASSVGSQSSAGSVGSSGVAATISTPVTIQNMTSSYVTITSHVL     1153
AFF4_Danio      N-KSAGMPSPVSPKLSPGSAGSYSS--SSSSQS-ASSSVTIPQRIHQMAASYVQVTSNFL     1024
AFF4_Xenopus    S-KSVSMPSPVSPKLSPGNSNNYSS--SVSSSSGSTSSVTIPQRIHQMAASYVQVTSNFL     1103
AFF4_MOUSE      S-KAVGMPSPVSPKLSPGNSGSYSS--GGSSASASGSSVTIPQKIHQMAASYVQVTSNFL     1101
AFF4_RAT        S-KAVGMPSPVSPKLSPGNSGSYSS--GGSSASASGSSVTIPQKIHQMAASYVQVTSNFL     1091
AFF4_HUMAN      S-KAVGMPSPVSPKLSPGNSGNYSS--GASSASASGSSVTIPQKIHQMAASYVQVTSNFL     1104
AFF4_BOVIN      S-KAVGMPSPVSPKLSPGNSGNYSS--GASSASASGSSVTIPQRIHHMAASYVQVTSNFL     1105
AFF2_HUMAN      NGKNTPSPVSLNN-VSPINAM----------GNCNNGPVTIPQRIHHMAASHVNITSNVL     1254
AFF3_HUMAN      SGKSTGTPSPMSPNPSPASSVGSQGSLSNASALSPSTIVSIPQRIHQMAANHVSITNSIL     1169
                                                                   H   H
                                                                   *

                     α6          α7                  α8
AFF1_HUMAN      TAFDLWEQAEALTRKNKEFFARLSTNVCTLALNSS-LVDLVHYTRQGFQQLQELTKTP-     1210
AFF4_Danio      YATEVWDQAEQLAKEQREFFTELDKAMGPLIFNTSSMTDLVRFTRQGLHWLRLDAKLIQ     1083
AFF4_Xenopus    YATEIWDQAEQLCKEQNDFFTELDKVMGPLIFNSSTMTELVRYTRQGLHWLRLDAKLK-     1161
AFF4_MOUSE      YATEIWDQAEQLSKEQKEFFAELDKVMGPLIFNASIMTDLARYTRQGLHWLRQDAKLIS     1160
AFF4_RAT        YATEIWDQAEQLSKEQKEFFAELDKVMGPLIFNASIMTDLARYTRQGLHWLRQDAKLIS     1150
AFF4_HUMAN      YATEIWDQAEQLSKEQKEFFAELDKVMGPLIFNASIMTDLVRYTRQGLHWLRQDAKLIS     1163
AFF4_BOVIN      YATEIWDQAEQLSKEQKEFFAELDKVMGPLIFNASIMTDLVRYTRQGLHWLRQDAKLIS     1164
AFF2_HUMAN      RGYEHWDMADKLTRENKEFFGDLDTLMGPLTQHSS-MTNLVRYVRQGLCWLRIDAHLL-     1311
AFF3_HUMAN      HSYDYWEMADNLAKENREFFNDLDLLMGPVTLHSS-MEHLVQYSQQGLHWLRNSAHLS-     1226
```

Figure S2

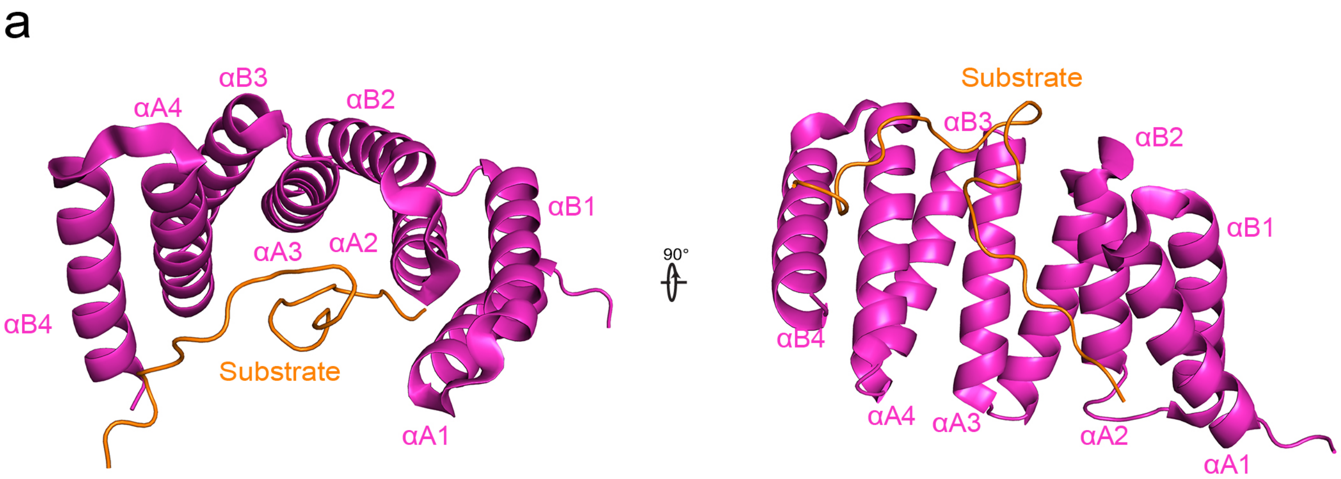
a
αB3
αA4
αB2
αB1
αA3
αA2
αB4
Substrate
αA1
90°
Substrate
αB3
αB2
αB1
αB4
αA4
αA3
αA2
αA1

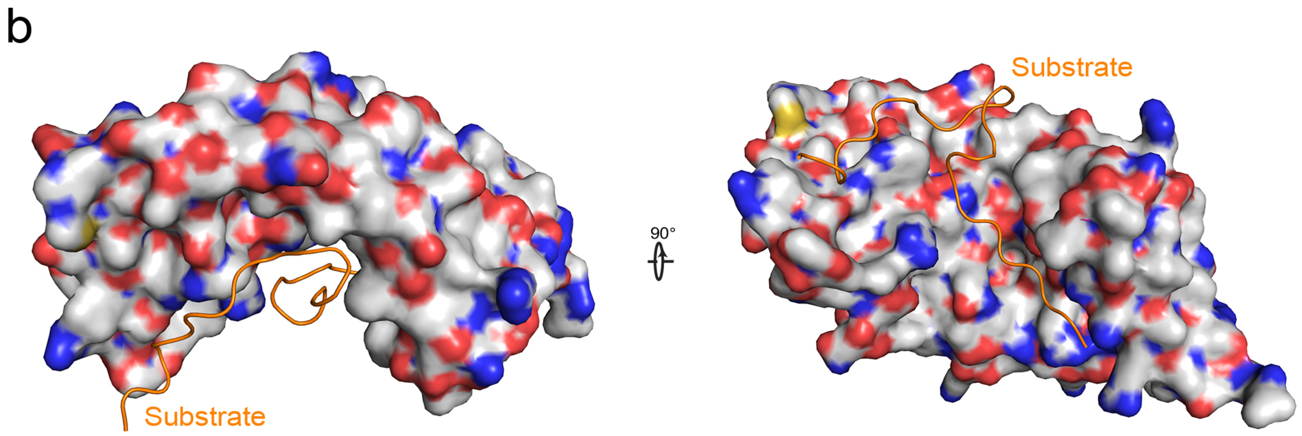
b
90°
Substrate
Substrate

Figure S3

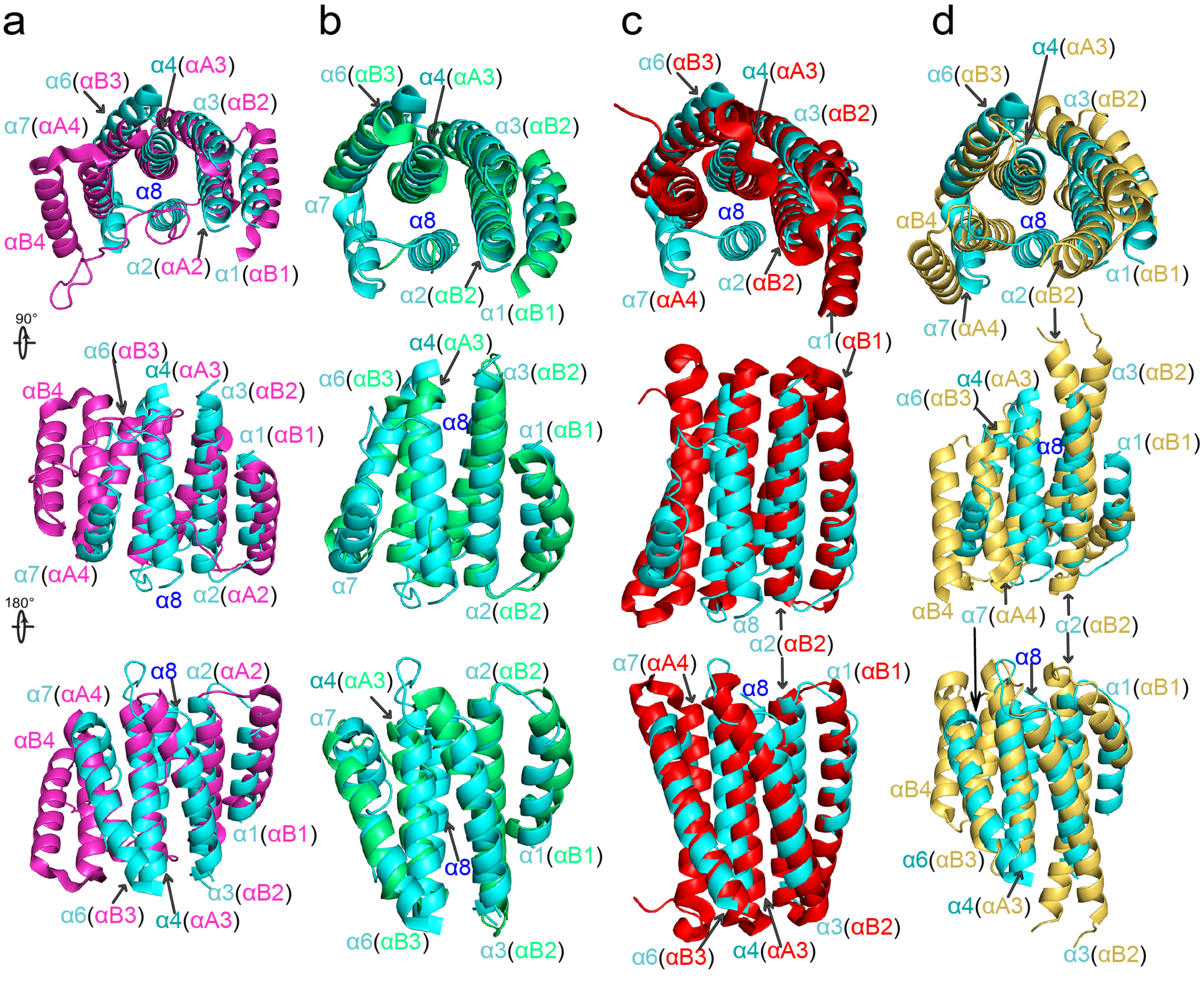
a
b
c
d
α6(αB3)
α4(αA3)
α3(αB2)
α7(αA4)
α8
αB4
α2(αA2)
α1(αB1)
α7
α2(αB2)
α1(αB1)
90°
180°
α8
α7(αA4)
α6(αB3)
α4(αA3)
α3(αB2)
αA2

Figure S4

Supplementary Figure Legends

Figure S1. Omit map of AFF4-THD.

The map corresponding to α4, α5 (a), and α6 (b) of AFF4-THD is displayed at a contour level of 1σ (magenta), with AFF4-THD shown in stick. R1012, F1014, and Y1096 are highlighted with labels.

Figure S2. Sequence alignment of AFF-THD domain.

Sequence alignment was carried out by Clustal Omega. HUMAN: *Homo sapiens*; Danio: *Danio rerio*; Xenopus: *Xenopus tropicalis*; Bovin: *Bos taurus*; Mouse: *Mus musculus*; Rat: *Rattus rattus.* The secondary structures are shown on top of the sequence in cyan. Key residues of Core1 are colored in blue, while those of Core2 are colored in red. Residues participating in both hydrophobic cores are colored in purple. "H" colored in magenta indicates the residues participate in hydrogen bonds discussed in this manuscript. "*" colored in green denotes the center residues of two hydrophobic cores.

Figure S3. Structural feature of TPR domain

a. Cartoon representation of TPRs is shown. The TPRs solenoid forms crescent sharp with Helix A locating in the concave side. TPR is shown in magenta while its substrate is shown in orange. Left: topview. Right: sideview. **b.** Surface model is shown here with same orientation as in (a). Afadin-TPR (5A6C-B) is used here as representative.

Figure S4. Comparison AFF4-THD to classic TPR domains

Superposition with (**a**) Afadin-TPR (5A6C-B), (**b**) APC-5(5g05-O), (**c**) 14-3-3 β6 (5ok9-B), and (**d**) Designed helical Repeat (5cwq-A), respectively. The molecules are colored in cyan,

magenta, green, red, and yellow, respectively. Upper: top-view. Middle, side-view. Bottom: reverse side-view to middle.

Supplementary Table S1.

Statistics of Crystallographic Data Reduction and Refinement

	Native	Se-Met
Data collection		
Space group	$I2_12_12_1$	$I2_12_12_1$
Unit cell parameters		
a, b, c (Å)	41.411, 79.762, 185.667	41.375, 80.986, 184.493
α, β, γ (°)	90.000, 90.000, 90.000	90.000, 90.000, 90.000
Wavelength (Å)	1.000	0.9798
Resolution (Å)	46.42-2.4 (2.485-2.4)	50.000-2.90 (3.00-2.90)
Unique reflections	12454 (1170)	7200(683)
Completeness (%)	99.38 (95.50)	99.5 (96.9)
Redundancy	19.0 (15.9)	12.6 (11.7)
Rsym	0.085 (0.535)	0.080(0.256)
<I>/<σ(I)>	28.03 (4.33)	30.47 (7.5)
$CC_{1/2}$	0.975	0.998
Refinement		
Resolution (Å)	46.42-2.4 (2.485-2.4)	
R_{work}/R_{free} (%)	21.35/26.70(29.58/44.38)	
Average *B*-factor (Å)	75.69	
R. m. s. deviation from ideality		
Bond length (Å)	0.009	
Bond angle (°)	1.06	
Ramachandran Plot (%)		
Favored	95.24	
Allowed	4.76	
Outliers	0	

Values in parentheses are for the highest resolution shell. R_{free} was calculated with 10% of the reflections selected randomly.

Supplementary Table S2.

Contribution of hydrophobic cores of AFF1/4-THD to dimerization

Constructs	Elution volume(ml)	Theoretical M.W.of monomer (kD)	Calculated M.W.(kD)
AFF4-WT	9.93	30.42	56.78
AFF4-F1014A	10.8	30.35	40.19
AFF4-Y1096A	10.98	30.33	37.42
AFF4-V1097A	10.55	30.4	44.38
AFF4-Y1096A-V1097A	11.02	30.3	36.83
AFF4-F1014A-Y1096A	11	30.26	37.12
AFF4-F1014A-V1097A	10.78	30.32	40.51
AFF4-F1014A-Y1096A-V1097A	11.03	30.23	36.68
AFF4-Y1096H	10.6	30.4	43.51
AFF4-1082-1086Δ(5G)	10	30.21	55.22
AFF4-Y1096F-T1100A	9.92	30.38	57.00
AFF4-F1103A	10.57	30.35	44.03
AFF4-Y1024A	9.98	30.33	55.66
AFF4-S1025A	9.93	30.41	56.78
AFF1-WT	9.65	31.73	63.46
AFF1-F1063A	9.99	31.65	55.44
AFF1-Y1145A	9.82	31.63	59.31
AFF1-F1063A-Y1145A	10.03	31.56	54.57

Alanine mutagenesis and analytical gel filtration are performed to check the contribution of hydrophobic cores as well as the only β-sheet in the structure to dimerization. The DNAs of AFF4 and AFF1 are 902-1163 and 953-1210 respectively. WT, wild type.1082-1086Δ(5G) means 1082-1086 of AFF4 are replaced with 5-glycine. Elution volume indicates the peak position of proteins in gel filtration. Theoretical M.W. of monomer indicates the accurate M.W. based on the amino acid sequence. Calculated M.W. indicates the molecular weight of target protein calculated according to the Standards.
